# A lightweight tunnel vehicle re-ldentification model based on YOLOv11n and FaceNet

**DOI:** 10.1371/journal.pone.0339450

**Published:** 2025-12-30

**Authors:** Wei Wang, Xu Liu, Yangguang Ye, Xianjin Xu, Minghui Wang, Zheng Zhang

**Affiliations:** 1 School of Mechanical Engineering, Hubei University of Technology, Wuhan, Province, China; 2 Key Lab Of Modern Manufacture Quality Engineering, Hubei University of Technology, Wuhan, Province, China; 3 China Railway Bridge Science Research Institute, Ltd, Wuhan, Province, China; Instituto Politecnico Nacional, MEXICO

## Abstract

With the advancement of computer vision, vehicle re-identification (Re-ID) in tunnel environments faces critical challenges like low-resolution imagery, lighting variations, and occlusions, which greatly limit the effectiveness of existing algorithms. This study presents a novel framework for intelligent tunnel vehicle monitoring, integrating lightweight detection and enhanced feature learning. Specifically, YOLOv11n is embedded as the front-end for lightweight detection; for vehicle Re-ID, the FaceNet model is optimized by replacing its Inception-ResNet backbone with MobileNetV3 and adding a Coordinate Attention module, along with a proposed joint loss function combining IoU-based hard triplet mining and Center Loss. A tunnel-specific dataset with 12,000 vehicle images is constructed, incorporating data augmentation to handle real-world surveillance complexities. Experimental results show: YOLOv11n achieves 98.63% mAP at 242 fps; the improved Re-ID model reaches 94.18% accuracy at 25.43 fps (0.81 GFLOPs, 3.51M params), outperforming baselines; ablation studies validate components, and AUC improves by 2.44%. This work provides a robust solution for real-time tunnel vehicle monitoring, with potential extensions to multi-modal fusion and cross-tunnel transfer learning.

## 1. Introduction

With the rapid advancement of computer vision technology, the informatization, efficiency, safety, and intelligence of road traffic systems have become the core orientation for the development of modern transportation infrastructure. As key objects of interest in road surveillance video data, vehicles have garnered extensive research attention in the field of computer vision, particularly within the sub-domains of recognition [1], detection [2], and classification [3]. In recent years, researchers have typically employed deep learning networks to extract discriminative vehicle features, followed by the deployment of feature matching strategies, thereby accomplishing the task of vehicle re-identification [4,5]. Notably, tunnel environments, as crucial components of urban road networks, impose unique challenges on vehicle monitoring attributed to their intrinsic structural and environmental properties. The fixed single camera angles, low-resolution image acquisition, random occlusions, and lighting fluctuations in tunnels result in insufficient effective information in the captured images and significant appearance variations of the same vehicle target. These issues necessitate the urgent development of a vehicle re-identification solution tailored to tunnel scenarios, which constitutes the primary motivation of this study.

In recent years, as a subfield of object re-identification tasks, vehicle re-identification has been subjected to extensive investigations by researchers in the field. Representative studies include the Pose Apprise Transformer network proposed by RiShi et al. [6], which integrates pose generation and local attribute classification to capture local vehicle features, and the multi-branch network developed by Chen et al. [7], which achieves global-local feature fusion through self-distillation. Other research efforts have focused on semantic feature extraction [8], local feature enhancement [9], attention mechanism integration [10,11], and self-supervised learning [12], while a subset of studies has explored traditional methods based on moments and Support Vector Machine (SVM) [13,14]. Despite the significant advancements achieved in recognition accuracy and dataset diversity within the field of vehicle re-identification, existing research still exhibits notable limitations. Most state-of-the-art algorithms are validated on general public datasets that lack tunnel-specific samples, failing to accommodate the unique challenges inherent in tunnel environments, such as constrained camera angles and severe appearance variations. Consequently, current models are incapable of effectively addressing vehicle re-identification tasks in tunnel scenarios, and there remains a dearth of dedicated datasets and lightweight, high-precision models for this specific application scenario.

To address the aforementioned challenges, this study constructs a tunnel-specific vehicle dataset and proposes an optimized vehicle re-identification model. Specifically, a comprehensive dataset is first established using vehicle samples collected from a surveillance system deployed in a tunnel in China. Through manual screening and data augmentation techniques, the diversity of the dataset is enhanced, resulting in a final dataset containing 12,000 valid vehicle images. Second, the FaceNet [15] network is improved by embedding the YOLOv11n [16] detection model into its front-end input stage, replacing the original Inception-ResNet [17] feature extraction backbone with the more lightweight MobileNet V3 [18]. Furthermore, to compensate for the limitations of MobileNet V3 in feature representation, a Coordinate Attention (CA) [19] module is introduced to enhance the feature extraction capability of the model. Additionally, to further improve the feature learning performance of the model, an Intersection over Union (IoU)-based strategy is adopted for the random selection of hard triplets, and Center Loss is integrated to construct a joint loss function. It is noteworthy that this study adopts an open-set recognition paradigm, wherein the vehicle samples in the test set are not encountered by the model during the training phase. The key contributions of this study are summarized as follows:

(1) A tunnel-specific vehicle dataset is constructed utilizing surveillance videos from a tunnel in China. Manual screening and data augmentation techniques are employed to improve the diversity of the dataset, resulting in a final dataset comprising 12,000 valid vehicle images.
(2) Through comparative experiments and validation on the self-constructed dataset, YOLOv11n is selected as the target detection model and integrated into the front-end of the subsequent vehicle re-identification model.
(3) Inspired by triplet loss, FaceNet is selected as the baseline recognition model. Its feature extraction backbone is reconstructed using the more lightweight MobileNet V3, and CA modules are introduced to enhance feature extraction performance. Moreover, an IoU-based mechanism is designed for hard triplet selection, and Center Loss is incorporated to address the limitation of triplet loss in failing to provide globally optimal constraints.

Finally, to verify the reliability and effectiveness of the proposed algorithm, ablation experiments are conducted, comparative analyses are performed against mainstream vehicle re-identification models respectively on the self-constructed dataset in this study and public datasets, performance curves (i.e., Receiver Operating Characteristic (ROC) curves and Cumulative Matching Characteristic (CMC) curves) are plotted based on the test set results, and similarity matrices are constructed for vehicles of typical categories.

## 2. Tunnel vehicle detection and re-identification model

### 2.1 Tunnel scene dataset

To verify the effectiveness of the proposed tunnel vehicle monitoring model, this study constructs a tunnel vehicle dataset using surveillance videos captured by two cameras installed at the entrance and exit of Pingyanshan Tunnel in China within a specific time window. After the initial screening process, the dataset initially contained 14,332 vehicle images. Subsequent manual filtering was performed to remove duplicate images across the two cameras, images with non-target obstructions, and images captured outside the predefined time period; this resulted in a preliminary cleaned dataset consisting of 9,458 images (covering both tunnel entrance and exit scenarios). In terms of vehicle type distribution, the dataset is categorized by vehicle model, with the ratio of trucks (including special large vehicles) to cars (excluding the distinction between fuel vehicles and new energy vehicles) being approximately 1:15. Regarding lighting conditions, the tunnel is equipped with sufficient and constant lighting facilities, ensuring stable lighting inside the tunnel; however, the area outside the tunnel is affected by natural light, leading to uneven lighting at the tunnel entrance-exit junction. Considering the common challenges of uneven lighting and camera jitter in real-world tunnel surveillance environments, targeted data augmentation operations were implemented on the preliminarily cleaned dataset: specifically, gradient attenuation was applied to mitigate the impact of uneven lighting, and Gaussian blur was added to simulate the effect of camera jitter. After completing these augmentation steps, the final dataset size reached 12,000 images in total. This finalized tunnel vehicle dataset was utilized to validate the effectiveness of the proposed algorithm model.

**Fig 1:**
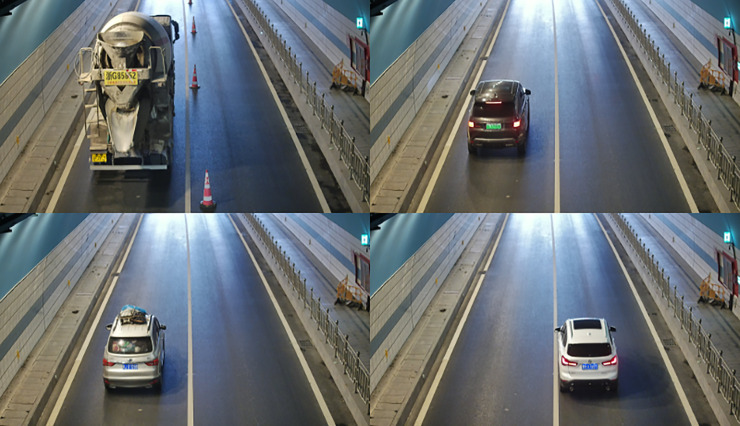
Representative samples from the dataset.

The constructed dataset was partitioned into training, validation, and test sets following an 8:1:1 ratio. Specifically, the training set included 9,600 images, the validation set contained 1,200 images, and the test set also comprised 1,200 images. To further en-hance the robustness of the model, distinct vehicle instances were assigned to each of the three subsets (i.e., no overlap of vehicle individuals across the training, validation, and test sets). Representative examples of images from the dataset are presented in Fig 1.

### 2.2 Tunnel vehicle detection model

The monitoring system model can be divided into two major frameworks: the vehicle detection framework and the vehicle re-identification framework. For the vehicle detection framework, given that object detection can be easily accomplished with current technology, this paper conducts a comprehensive comparison of mature open-source lightweight object detection models to perform the detection task. For the vehicle re-identification framework, which presents significant challenges, ordinary re-identification models are unable to effectively meet the task requirements. Therefore, this paper performs algorithmic structure optimization based on the FaceNet algorithm, specifically tailored for tunnel scenarios. Below, we will provide a detailed introduction to the specific content and research work related to these two frameworks in this paper.

#### 2.2.1 Lightweight vehicle detection based on YOLOv11n.

In the field of object detection, deep learning-based algorithms are primarily classified into two categories: one-stage detectors and two-stage detectors, with the YO-LO [20] series and RCNN [21] series serving as their respective representative frame-works. One-stage detectors exhibit distinct advantages, including high detection speed, reduced susceptibility to background-related errors, and the ability to learn generalized object feature representations; however, they are limited by relatively low accuracy (in terms of localization precision and detection rate) and inferior performance in small object detection tasks. In contrast, two-stage detectors demonstrate strengths in high accuracy (localization precision and detection rate), mature anchor-based mechanisms, and efficient shared computational burdens; yet, they are hindered by slow inference speed, prolonged training cycles, and relatively high false positive rates.

Considering the characteristics of tunnel scenarios—such as a single type of detection target, fixed single viewing angle, limited computational resources of edge devices, and the critical requirement for real-time detection—this study prioritizes lightweight architecture design and high frame rate performance for the vehicle detection module. Meanwhile, considering that the widespread application of the YOLO series is highly compatible with subsequent task deployment, the YOLO series was selected as the base framework for vehicle detection. Specifically, the nano versions of YOLOv5 [22], YOLOv8 [23], YOLOv10 [24] and YOLOv11 models were compared on the self-constructed tunnel vehicle dataset. The comparison encompassed their performance under both default (original) settings and the custom dataset conditions (all tests were conducted on an RTX 3090 Ti GPU), with evaluation metrics including mean Average Precision (mAP), model parameter count, and inference speed (tested on CPU and the official NVIDIA A100 GPU, respectively). The detailed comparison results are presented in Table 1.

**Table 1:** Model parameter comparison.

Model	Map	Params (M)	Speed (CPU)	Speed (GPU)	Map (OUR)	Speed (OUR)
**YOLOv5n**	28	2.6	73.6ms	1.12ms	88.74%	3.73ms
**YOLOv8n**	37.3	3.2	80.4ms	1.47ms	97.43%	4.06ms
**YOLOv10n**	38.5	2.3	--	1.56ms	98.47%	4.22ms
**YOLOv11n**	39.5	2.6	56.1ms	1.5ms	98.63%	4.12ms

As presented in Table 1, YOLOv8n, YOLOv10n, and YOLOv11n all meet the preset performance requirements in terms of mAP and inference speed. Among these candidates, YOLOv10n stands out with the lightest weight (2.3M parameters) and a competitive mAP of 38.5, while its GPU inference speed (1.56ms) and optimized performance on the custom dataset (Map(OUR) = 98.47%) are also commendable. However, YOLOv11n outperforms it in comprehensive performance: with a 4.2% higher baseline mAP (39.5 vs. 38.5), a slightly faster GPU inference speed (1.5ms vs. 1.56ms), and a more balanced optimized performance (Map(OUR) = 98.63%, Speed(OUR) = 4.12ms). Additionally, compared to YOLOv8n, YOLOv11n achieves a 23.1% reduction in parameter count—this advantage in model lightweighting, combined with its superior accuracy and speed, solidifies its selection as the baseline algorithm for the vehicle detection module in this study. Specifically, YOLOv11n delivers an inference speed of approximately 242 frames per second (fps), which is substantially higher than the 30 fps frame rate of the tunnel surveillance cameras. This significant performance margin ensures that the detection module fully meets the real-time requirements of practical tunnel monitoring scenarios.

#### 2.2.2 Optimization of detection for restricted scenarios.

In practical tunnel monitoring scenarios, due to the fixed installation position of surveillance cameras, the images in the constructed dataset are predominantly cap-tured from a downward-inclined viewing angle. Under this perspective condition, if detection models are only used to detect entire vehicle regions, two key issues arise: first, it fails to generate input images with unified standards for the subsequent vehicle re-identification module; second, it inevitably introduces low-contribution vehi-cle-related features (e.g., irrelevant background clutter) into the feature extraction process. Both issues collectively lead to significant degradation in the efficiency of the subsequent re-identification model and its ability to extract discriminative features.

**Fig 2:**
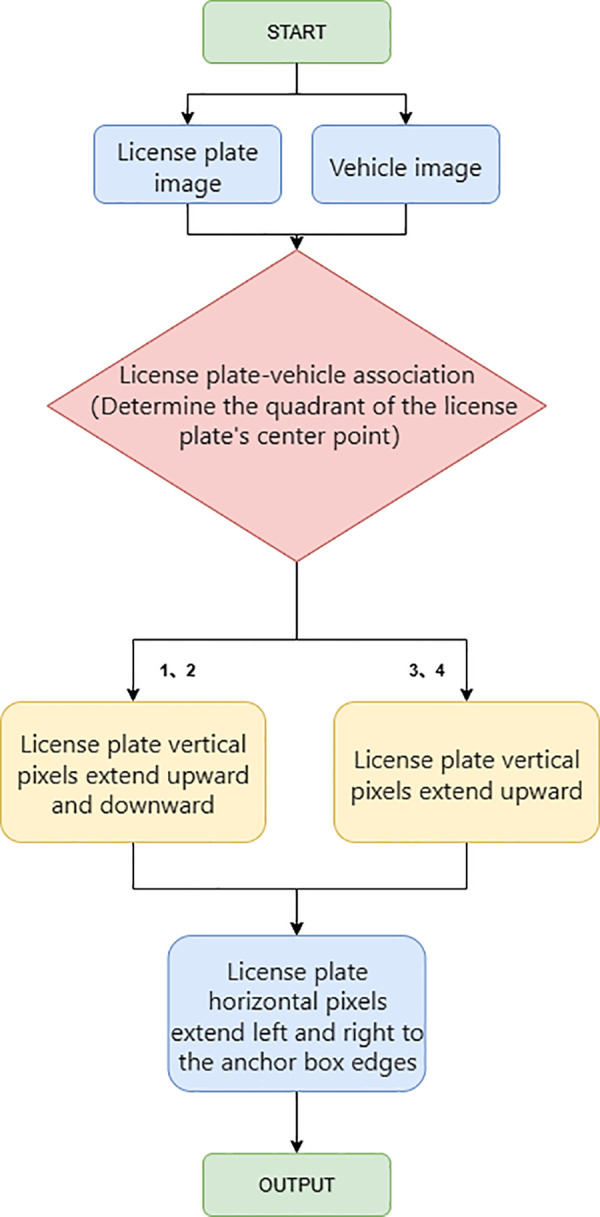
Detection optimization flow chart.

**Fig 3:**
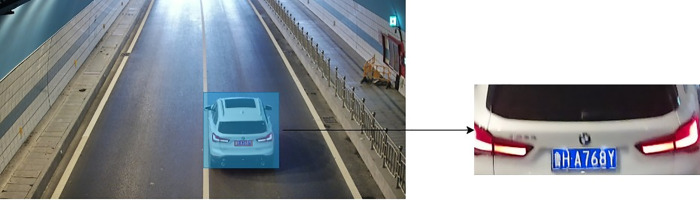
Detection optimization explanatory diagram.

To mitigate the aforementioned problems, this study proposes integrating a li-cense plate localization module into the existing vehicle detection pipeline. Specifically, by exploiting the inherent spatial correlation between license plates and the bounding boxes of detected vehicles, the rear-view (tail-side) images of vehicles— which contain most of the critical discriminative features of vehicles—are accurately extracted. This optimization strategy not only ensures that the input images for the vehicle re-identification module conform to consistent specifications but also effectively sup-presses the interference of background noise. The flow chart of the detection optimization process and the corresponding explanatory diagram are presented in Figs 2 and 3, respectively.

### 2.3 Vehicle re-identification based on the FaceNet model

#### 2.3.1 Initial FaceNet framework.

**Fig 4:**
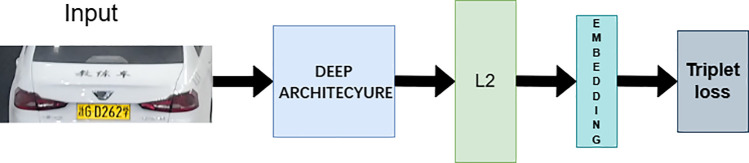
Architectural schematic of FaceNet framework.

The vehicle re-identification framework in this paper is based on the FaceNet model, and its structure is illustrated in the Fig 4 below.

As depicted in Fig 4, the original FaceNet model processes input target images via the DEEP ARCHITECTURE backbone layer, which conducts deep feature extraction and dimensionality reduction to generate feature vectors. The extracted image feature vectors are subjected to L2 normalization to avoid the impact of feature magnitude imbalance on subsequent similarity comparisons. Finally, triplet loss is used to compute the model’s training loss, and this loss value guides backpropagation to complete the model training process. For similarity comparison, the Euclidean distance between the final feature vectors is calculated, and whether the two targets are the same is determined by comparing the computed Euclidean distance with a predefined threshold.

Nevertheless, in the tunnel scenarios focused on in this study, challenges such as uneven lighting, vehicle appearance variations, and motion blur caused by camera jitter render the original FaceNet model’s feature extraction capability, network structure, and discrimination methods insufficient for vehicle re-identification. Therefore, this study proposes two key improvements:

(1) reconstructing a lightweight backbone network and integrating a spatial attention CA module;
(2) designing a hard triplet selection mechanism and incorporating Center Loss to construct a joint loss function.

These enhancements are targeted at improving feature extraction accuracy, enhancing model robustness, and boosting the overall performance of the system in the challenging tunnel environment. The details of these improvements are elaborated in the following sections..

#### 2.3.2 Feature extraction backbone network.

The original FaceNet model employs Inception-ResNet-v1 as its backbone network, whose core modules—parallel multi-scale convolutions and residual connections—significantly enhance network accuracy and convergence. However, its drawbacks, including a large parameter size (typically over 10 million), high FLOPs, slow inference speed, and excessive memory consumption, make it unsuitable for deployment on edge or embedded devices for real-time mobile tasks. To address this, this paper replaces the backbone network with MobileNetV3.

MobileNetV3 significantly reduces computational complexity and parameter count while maintaining strong feature extraction capabilities through the h-swish activation function, depthwise separable convolutions, and the efficient SE (Squeeze-and-Excitation) [25] attention module.

Since this study aims to achieve a systematic tunnel vehicle monitoring system, it must balance extremely high accuracy with real-time inference performance. Therefore, the FaceNet backbone Inception-ResNet-v1 is reconstructed into a lightweight MobileNetV3. However, MobileNetV3, designed for highly lightweight mobile scenarios, lacks sufficient feature coverage in its network structure, leading to poor expressive power under nonlinear conditions. To mitigate this, the model architecture is optimized and adjusted.

**Fig 5:**
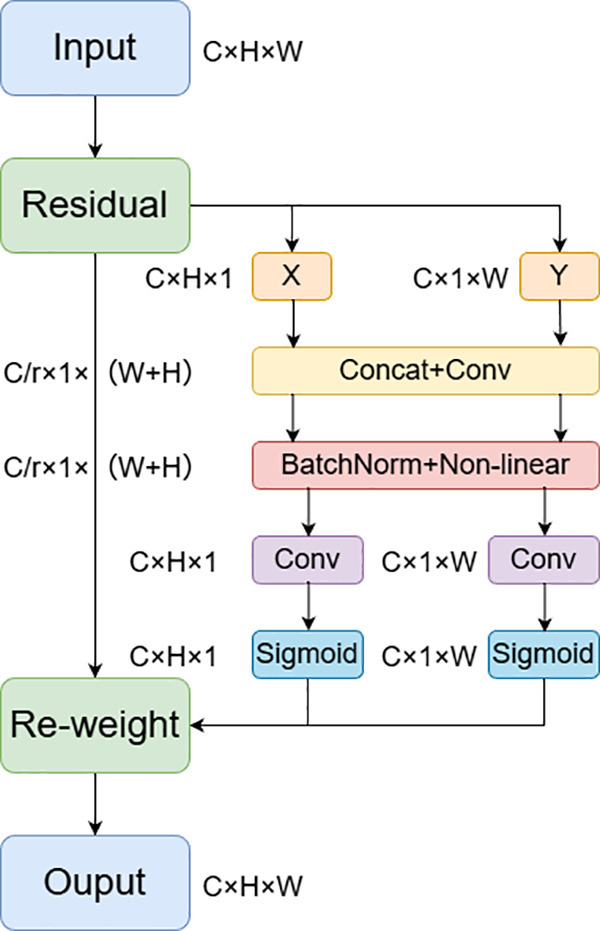
Structural diagram of coordinate attention module.

The original network extensively uses the SE (Squeeze-and-Excitation) module in inverted residual blocks to enhance feature representation through channel attention. However, SE has limited ability to capture spatial relationships. To address the spatial sensitivity of critical vehicle components (e.g., headlights, license plates), this paper introduces the Coordinate Attention (CA) module. The mathematical principle of CA involves decomposing attention mechanisms into coordinate spaces, separately computing attention weights for rows and columns, and then fusing the results. This approach enables CA to enhance spatial feature weighting for complex tasks without excessive computational overhead, compensating for SE’s overemphasis on channel weights and neglect of spatial relationships. The structure is illustrated in Fig 5.

Initially, the input feature map undergoes horizontal pooling and vertical pooling, respectively. The corresponding formulas are presented as follows [19]:


$$F_{h}(c,h)=\frac{\text{1}}{W}\sum w=1WF(c,h,w)$$ (1)

$$F_{w}(c,w)=\frac{\text{1}}{H}\sum h=1HF(c,h,w)$$ (2)

Where $F_{h}(c,h)$ and $F_{w}(c,w)$ represent the horizontal and vertical pooled features of input F(c,h,w), which denotes the feature of the input image, respectively.

Subsequently, feature transformation is conducted to generate the coordinate attention map, with the corresponding mathematical principle presented as follows:


$$U_{h}=\sigma (BN_{h}(W_{h}*F_{h}+b_{h}))$$ (3)

$$U_{w}=\sigma (BN_{w}(W_{w}*F_{w}+b_{w}))$$ (4)

$$M(c,h,w)=U_{h}(c,h)⊗U_{w}(c,w)$$ (5)

Where σ denotes the sigmoid activation function; $BN_{h}$ and $BN_{w}$ represent the batch normalization layers for normalizing features in the horizontal and vertical directions, respectively; $W_{h}$ and $W_{w}$ stands for the dimension-reduction convolution kernels; $b_{h}$ and $b_{w}$ are the convolution bias terms; and M(c,h,w) is the finally generated coordinate attention map.

Finally, feature weighting is performed to obtain the final output features, with the corresponding mathematical principle presented as follows:


$$F^{\prime }=F⊗M$$ (6)

Where F represents the original input feature; M denotes the coordinate attention map obtained from the aforementioned formula; and $F^{\prime }$ stands for the feature finally output after enhancement by the CA attention module.

**Fig 6:**
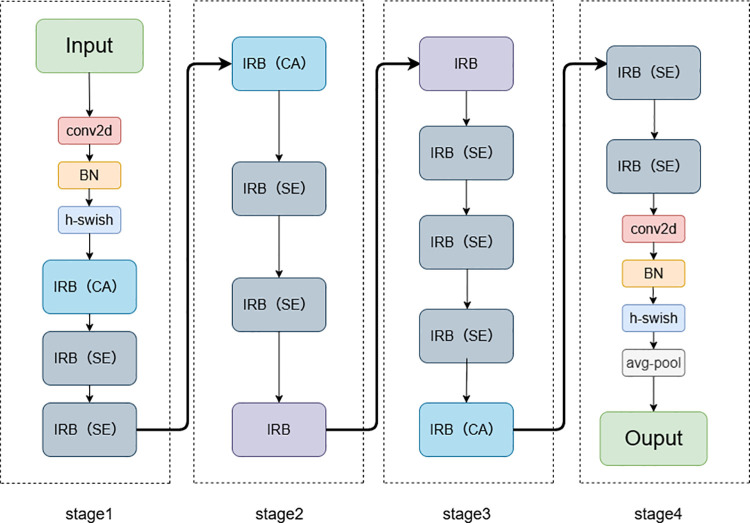
Optimized MobileNetV3 backbone network.

The final backbone architecture of the proposed network is illustrated in the Fig 6 below:

#### 2.3.3 Loss function configuration.

**Fig 7:**
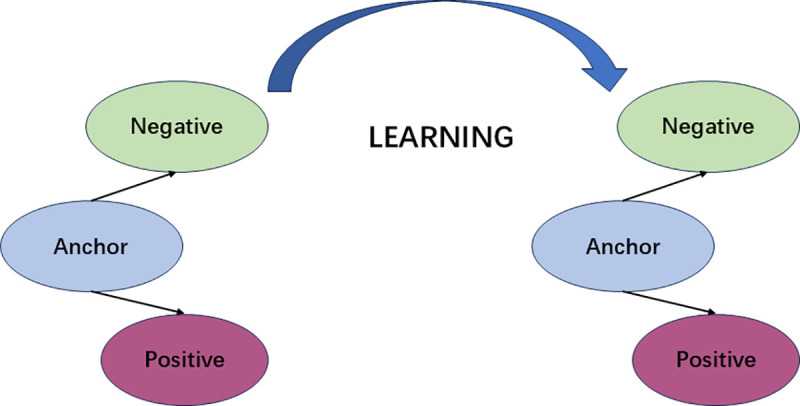
Triplet loss strategy visualization.

In the original FaceNet model, the triplet loss function optimizes the discriminative power of the feature embedding space by constructing contrasting relationships among an anchor sample, a positive sample, and a negative sample. This is illustrated in the Fig 7 below.

The overall triplet loss is calculated using the following formula [15]:


$$L_{triplet}=\sum_{i=1}^{N}\left[∥f(x_{i}^{a})-f(x_{i}^{p}){∥}_{2}^{2}-∥f(x_{i}^{a})-f(x_{i}^{n}){∥}_{2}^{2}+\alpha \right]+$$ (7)

Where $∥\cdot {∥}_{2}^{2}$ stands for the Euclidean distance function, $∥f(x_{i}^{a})-f(x_{i}^{p}parallel_{2}^{2}$ and $∥f(x_{i}^{a})-f(x_{i}^{n}parallel_{2}^{2}$ are the Euclidean distances between the anchor sample and the positive sample, and between the anchor sample and the negative sample, respectively. The term α is a margin hyperparameter that defines the minimum separation required for the triplet loss, and in this study, it was set to 0.25.

Although the core concept of triplet loss is beneficial for enhancing the model’s feature capture capabilities, it suffers from two primary limitations:

(1) Significant drawbacks in traditional random sampling: The random selection strategy exhibits notable shortcomings. On the one hand, the dominance of easy samples can lead the model into a local optimum prematurely. On the other hand, the inadvertent selection of mislabeled (noisy) samples can disrupt the geometric structure of the embedding space, thereby weakening the model’s ability to capture discriminative features.
(2) Neglect of absolute distance differences: Traditional triplet loss relies solely on the difference between the Euclidean distances of the anchor sample to the positive and negative samples, ignoring their absolute values. Furthermore, due to the local nature of individual triplets, this loss formulation lacks a global constraint ensuring optimal separation across all data. Consequently, in certain scenarios, this can lead to the catastrophic failure where inter-class distances become smaller than intra-class distances, significantly compromising model performance.

To address these two major limitations, this paper proposes two key improvements: (1) a modified triplet loss function based on the Intersection over Union (IoU) principle, and (2) a combined loss function incorporating Center Loss.

(1) IoU-based Hard Sample Mining for Triplet Selection: The effectiveness of triplet training is directly proportional to the difficulty level of the triplets. Therefore, we leverage the IoU concept to quantify the difficulty of both easy samples and noisy samples. Specifically, triplets are selected to maximize their “hardness” by comparing the feature vector IoU between positive and negative samples. This IoU-based hard mining strategy is defined mathematically as follows:

$$IOU=\frac{A\cap B}{A\cup B}$$ (8)

Where A and B represent the regions of the target objects being compared, respectively.

The selection of hard samples is governed by the following mathematical representation:


$$\mu_{p}=\frac{\text{1}}{k}\sum {\mathrm{\backslash nolimits}}_{i=1}^{k}IoU(A,P_{i})$$ (9)

$$\mu_{n}=\frac{\text{1}}{m}\sum {\mathrm{\backslash nolimits}}_{j=1}^{m}IoU(A,N_{j})$$ (10)

$$P*=\underset{P_{i}}{argmin}(f(A,P_{i})-\mu_{p})$$ (11)

$$N*=\underset{N_{j}}{argmin}(f(A,N_{j})-\mu_{n})$$ (12)

$$P=P^{*}$$ (13)

$$N=N^{*}$$ (14)

Where $P_{i}$ and $N_{j}$ denote the sets of candidate positive samples and candidate negative samples, respectively; k and m represent their corresponding cardinalities ($P_{i}$ and $N_{j}$); $\mu_{p}$ and $\mu_{n}$ correspond to the mean IoU values computed across all samples in $P_{i}$ and $N_{j}$; and $P^{*}$ and $N^{*}$ are the samples closest to these mean IoU values within their respective sets, thus constituting the selected positive and negative samples. $P^{*}$ and $N^{*}$ are the samples closest to the mean IoU of their respective sets, P and N are the finally selected positive and negative samples, respectively.

(2) Integration of Center Loss for Intra-Class Compactness Enhancement: The core principle of Center Loss is to learn a unique feature centroid for each semantic class (e.g., different vehicle classes in tunnel datasets). It addresses the limitations of triplet loss by imposing a penalty on the deviation between the deep feature representation of each sample and the centroid of its corresponding class. This mechanism explicitly constrains feature embeddings to cluster around their class-specific centroids in the learned feature space, thereby achieving more compact intra-class feature distribu-tions. In large-scale tunnel vehicle datasets, frequently occurring vehicle classes often exhibit significant appearance variations—even for the same physical vehicle—due to factors such as varying lighting conditions, camera perspectives, and partial occlu-sions. In such scenarios, triplet loss may generate excessively high loss values, as the feature distance between anchor-positive sample pairs (which should theoretically be small) becomes unexpectedly large. To mitigate this issue and enhance the stability of model training under appearance variations, this study integrates Center Loss into the loss function framework. The mathematical formulation of Center Loss is defined as follows [26]:

$$L_{center}=\frac{\text{1}}{\text{2}}\sum_{j=1}^{B}{\left\|f_{t_{j}}-c_{y_{j}}\right\|}_{2}^{2}$$ (15)

Where $L_{center}$ represents the Center Loss value, $f_{t_{j}}$ denotes all deep feature vectors in the current batch, $c_{y_{j}}$ signifies the feature centroids corresponding to each semantic class, and B is the batch size of the current training iteration.

Crucially, during training, the Center Loss dynamically records feature centroids for each class at the mini-batch level to obtain corresponding class feature centers, minimizing distances between all features and their respective centroids.

By penalizing the deviation between samples of each class and their corresponding class centroids, Center Loss enhances the generalization capability and discriminative power of learned features. This method aims to aggregate samples of the same class as densely as possible, thereby improving feature compactness and separability. When employing joint supervision, it simultaneously maximizes both compactness and separability of samples.

The final loss function is expressed as follows:


$$L_{loss}=\lambda L_{triplet}+(1-\lambda )L_{center}$$ (16)

Where $L_{loss}$ represents the total combined loss function of the proposed network, $L_{triplet}$ denotes the modified triplet loss (IoU-based hard sample mining triplet loss) proposed in this study, $L_{center}$ signifies the Center Loss integrated for intra-class compactness enhancement. And λ denotes the weight parameter. In this study, λ is set to 0.90 after extensive experimental validation.

Through joint supervision with our proposed hard triplet loss and Center Loss, we not only effectively mitigate the sample selection limitations within triplets to achieve hard triplet training, but also avoid the inherent triplet limitation where inter-class distances may become smaller than intra-class distances. Consequently, this joint supervision approach better optimizes model representation and enhances the model’s capability to capture critical features.

**Fig 8:**
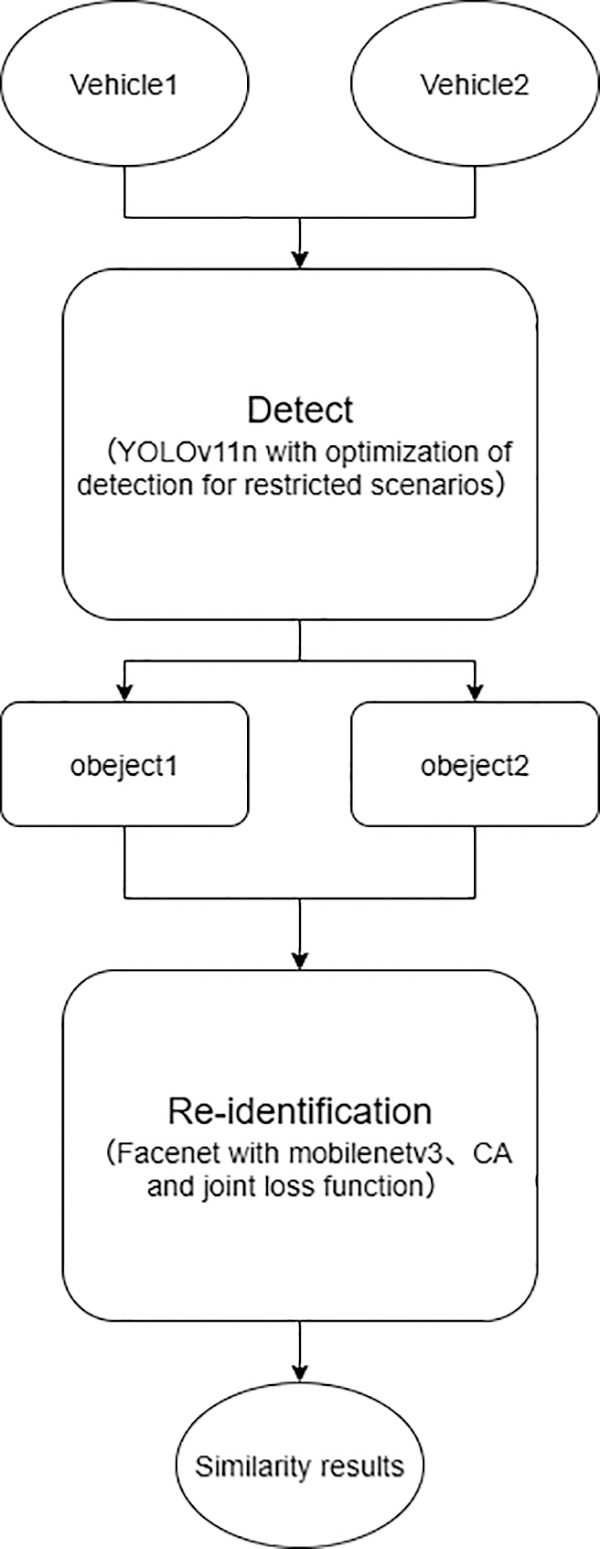
Overall algorithm flow chart.

Finally, the overall algorithm flow chart is shown in the following Fig 8.

As illustrated in the aforementioned flowchart, two vehicles first undergo the detection stage. Subsequent to this detection process, the resulting data of the two vehicles are fed into the re-identification (Re-ID) stage, where a similarity comparison is performed to determine whether they are the same vehicle. Specifically, both the vehicles at the tunnel entrance and those at the tunnel exit undergo the aforementioned detection stage separately. Following this detection, similarity comparisons are conducted between the entrance and exit vehicle data, which yields a similarity comparison matrix. Finally, by setting an appropriate threshold and leveraging this similarity comparison matrix, a judgment is made to confirm if the compared vehicles are identical.

## 3. Experimental results and analysis

### 3.1 Evaluation metrics

To comprehensively assess the performance of the proposed model from three critical dimensions—processing speed, computational accuracy, and deployment feasibility—this study adopts six core evaluation metrics: frames per second (FPS, for speed assessment), accuracy (ACC, for overall matching accuracy assessment), Precision (for measuring the proportion of true positives among predicted positives, evaluating matching correctness), Recall (for measuring the proportion of true positives successfully identified by the model, evaluating matching completeness), F1-score (for balancing Precision and Recall to reflect the model’s overall classification performance), computational complexity (measured in GFLOPs, for computational cost assessment), and model parameter size (measured in Params, for deployment feasibility assessment). The formal mathematical formulations of FPS, ACC, Precision, Recall, and F1-score are defined as follows:


$$Fps=\frac{T}{S}$$ (17)

$$Acc=\frac{A_{True}}{A_{True}+A_{False}}$$ (18)

$$Precision=\frac{TP}{TP+FP}\times 100\%$$ (19)

$$Recall=\frac{TP}{TP+FN}\times 100\%$$ (20)

$$F1-score=2*\frac{Precision*Recall}{Precision+Recall}$$ (21)

Where T denotes the total number of vehicles processed within a specified time duration; S represents the elapsed processing time in seconds; and $A_{True}$ and $A_{False}$ correspond to the counts of correctly matched and incorrectly matched vehicle pairs in the re-identification task, respectively. TP (True Positive) and FP (False Positive) correspond to the counts of correctly matched vehicle pairs and incorrectly matched vehicle pairs in the re-identification task, respectively; FN (False Negative) denotes the count of actually matched vehicle pairs that are incorrectly identified as unmatched by the model;

FPS, ACC, Precision, Recall, and F1-score directly measure the operational efficacy of the model, with FPS quantifying processing speed, ACC evaluating overall matching accuracy, Precision gauging the reliability of positive predictions (higher values mean fewer false matches between distinct vehicles), Recall assessing the completeness of positive detection (higher values indicate fewer missed valid matches), and F1-score serving as a balanced metric integrating Precision and Recall to comprehensively reflect the model’s classification performance; higher values for all these metrics indicate superior task-specific performance. GFLOPs expresses the total floating-point operations required for a single forward pass, reflecting computational complexity, while Params defines the total count of trainable weights in the neural architecture, and both metrics characterize the model’s structural complexity and serve as intuitive indicators of computational resource requirements and deployment feasibility. For models achieving comparable performance in terms of FPS, ACC, Precision, Recall, and F1-score, lower values of GFLOPs and Params are preferable as they signify enhanced model lightweightness.

### 3.2 Experimental setup

#### 3.2.1 Experimental platform setup.

The hardware and software configuration of the deep learning experimental platform employed in this study is detailed in Table 2.

**Table 2:** Test environment for research.

Name	Specific model
**CPU**	Intel Core I9-12900K
**GPU**	NVIDIA RTX 3090Ti (24g)
**RAM**	128G
**Operating system**	Windows10
**Software**	Pycharm(2024.3)
**Programming language**	Python(3.9.6)
**CUDA**	11.8
**Pytorch**	2.1.2

#### 3.2.2 Experimental parameter settings.

The hyperparameters for network training were configured as follows: the batch size was set to 30; the maximum learning rate and minimum learning rate were con-figured as 0.001 and 1 × 10 ⁻ ⁵, respectively (using scientific notation for consistency in numerical expression); the Adaptive Moment Estimation (Adam) optimizer was adopted for model optimization, with the momentum parameter set to 0.9; and a co-sine annealing learning rate scheduling strategy was employed to dynamically update the learning rate during training. For this study, a single training epoch is defined as one complete iteration over the entire training-validation dataset. After 300 consecutive training epochs (with the total loss function minimized iteratively), the optimal set of network weights was obtained.

### 3.3 Ablation experiment

To empirically validate the effectiveness of the proposed improvements, six comparative experimental groups were established with detailed parameter configurations specified in Table 3.

**Table 3:** Ablation experiment module configuration table.

Name	Mobilenetv3	CA	Loss
**1**	×	×	×
**2**	√	×	×
**3**	√	√	×
**4**	×	×	√
**5**	√	×	√
**6**	√	√	√

The comparative experimental results are presented in Table 4.

**Table 4:** Analysis of ablation experimental results table.

Name	Fps	ACC	Recall	Precision	F1-score	GFLOPS/G	Params/M
1	24.09	93.36%	95.90%	97.24%	96.57%	2.85	22.79
**2**	25.61	92.59%	95.19%	97.14%	96.15%	0.81	3.48
**3**	25.58	93.17%	95.75%	97.19%	96.46%	0.81	3.51
**4**	24.01	94.07%	96.61%	97.28%	96.94%	2.85	22.79
**5**	25.49	93.78%	96.33%	97.25%	96.79%	0.81	3.48
**6**	25.43	94.18%	96.48%	97.54%	97.00%	0.81	3.51

Ablation studies demonstrate that replacing the backbone network with MobileNetV3 significantly enhances inference speed, yielding a 6.31% FPS improvement over the baseline model while substantially reducing both GFLOPs and Params—albeit with marginal decreases in accuracy, Recall, Precision, and F1-score. Subsequent integration of the Convolutional Attention (CA) module slightly reduced FPS but increased accuracy by 0.58%, accompanied by improvements of 0.61% in Recall, 0.06% in Precision, and 0.33% in F1-score, with only minimal increments in model complexity. Adopting our proposed loss function further improved accuracy by 0.71% without compromising FPS or computational complexity, while additionally boosting Recall by 0.73%, Precision by 0.34%, and F1-score by 0.54%. After holistically balancing speed (FPS), accuracy, Recall, Precision, F1-score, computational overhead (GFLOPs), and model size (Params), the final architecture achieves a 5.56% FPS gain (25.43 fps), a 0.82% accuracy increase (94.18%), a 0.57% Recall improvement (96.48%), a 0.25% Precision enhancement (97.54%), and a 0.46% F1-score rise (97.00%) compared to the original model. Meanwhile, GFLOPs and Params are drastically reduced to 0.81G and 3.51M, respectively. These results reliably validate the efficacy of our architectural refinements across multiple performance metrics.

**Fig 9:**
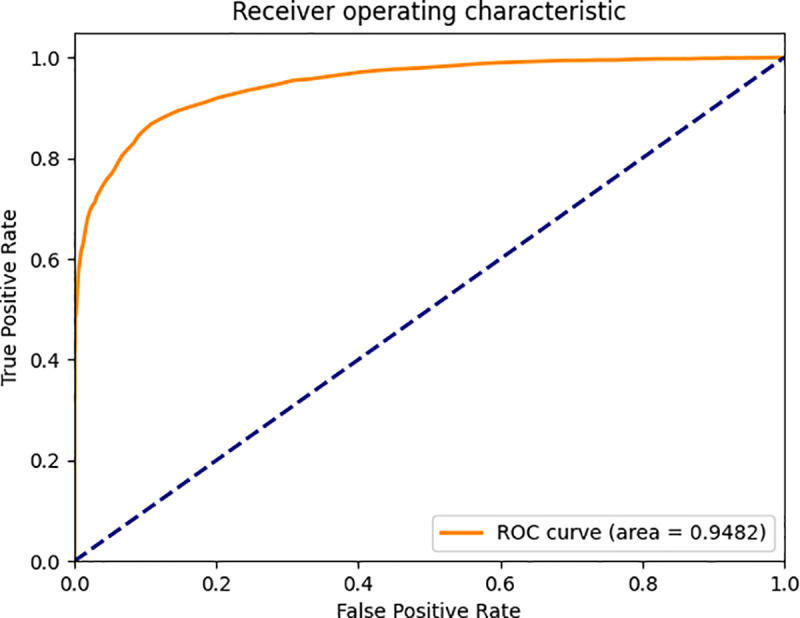
ROC curve of the original FaceNet model.

**Fig 10:**
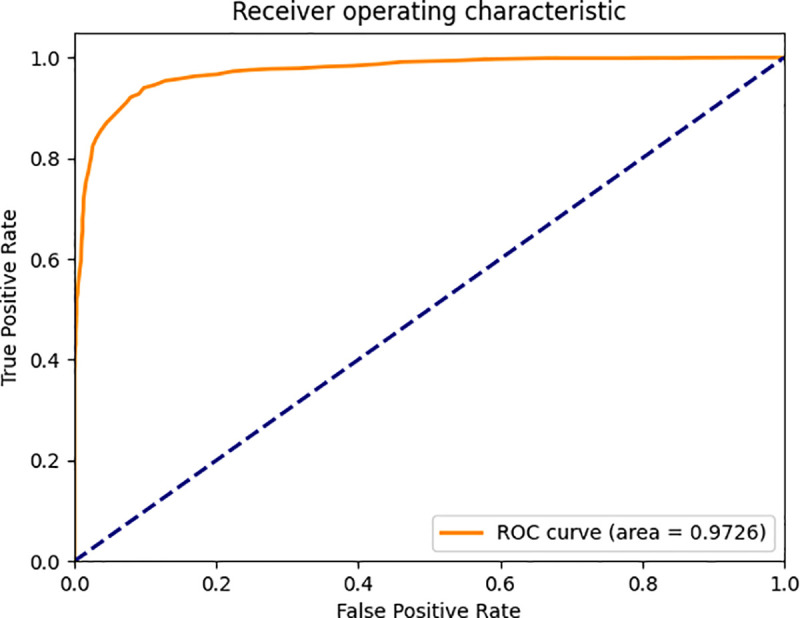
ROC curve of the improved model.

**Fig 11:**
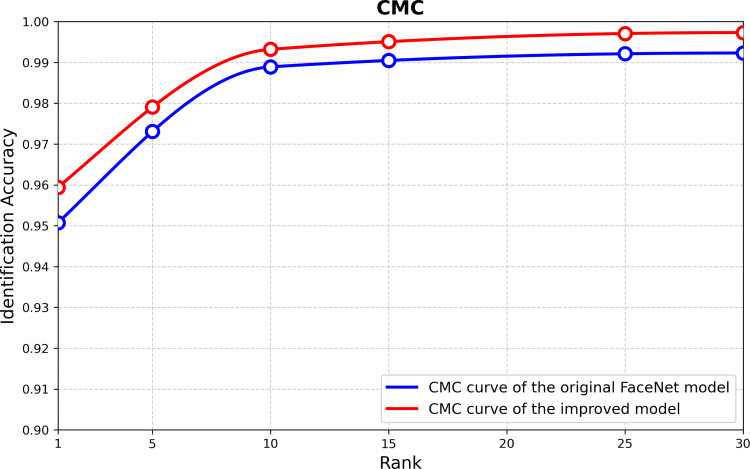
CMC curves of original and improved FaceNet models.

To provide an intuitive visual comparison of model performance, this study utilizes Receiver Operating Characteristic (ROC) curves and Cumulative Matching Characteristic (CMC) curves for analysis. The ROC curve illustrates the trade-off between the True Positive Rate (TPR) and False Positive Rate (FPR) under different classification thresholds: by adjusting the decision threshold for positive identification, a series of discrete data points can be generated on the ROC plane, which collectively form the ROC curve. In an optimal scenario, the ROC curve approaches the upper-left corner of the coordinate graph, with its Area Under the Curve (AUC) converging toward 1.0—indicating that the model maintains a high TPR and low FPR across various threshold settings, demonstrating excellent classification performance. The CMC curve, or Cumulative Matching Characteristic curve, represents the probability that the model achieves the first correct match. Its x-axis denotes the number of matching attempts, while the y-axis represents the average accuracy. Ideally, the CMC curve should be as close to the upper-left corner as possible, as it reflects the efficiency of the model in achieving accurate matching. The ROC curves comparing the baseline FaceNet model with our proposed framework are shown in Figs 9 and 10, and the comparative CMC curves are presented in Fig 11.

As evidenced in Figs 9 and 10, the AUC increased from 94.82% to 97.26% - a 2.44 percentage point improvement. Our refined curve demonstrates significantly closer convergence toward the ideal upper-left corner compared to the baseline. This visually confirms the performance superiority of our modified architecture over the original implementation, providing further validation for the effectiveness of our methodological enhancements.

As illustrated in Fig 11, the proposed model achieves an accuracy of 95.94% at Rank-1, and the overall trend of its CMC curve exhibits higher accuracy compared to the baseline model. Compared with the baseline, the CMC curve of our proposed model is closer to the upper-left corner in the ideal scenario. This visualization result intuitively confirms the performance superiority of the improved architecture over the original model, further validating the effectiveness of our methodological enhancements.

### 3.4 Comparative experiment

To further validate the efficacy of our proposed model, comparative experiments were conducted between our method and four baseline algorithms—original FaceNet, CosFace [27], ArcFace [28], and CenterFace [29]—on both our self-constructed dataset and the public VeRi776 [30] dataset. Specifically, CosFace enhances feature discrimination by incorporating a cosine similarity loss that maximizes the angular separation between classes in the embedding space. ArcFace extends this approach with additive angular margin optimization, further improving inter-class separability for face recognition compared to the conventional softmax loss. CenterFace employs a central alignment mechanism to boost detection accuracy through geometrically constrained localization. All baseline models were trained under the same hyperparameters and hardware conditions as our method. Quantitative comparisons of FPS, ACC, and Params are presented in Table 5.

**Table 5:** Comparative experimental results table.

Name	Fps	ACC	Fps (VeRi776)	ACC(VeRi776)	Params/M
**Facenet**	24.09	93.36%	23.47	84.26%	22.79
**Cosface**	24.38	91.74%	23.86	85.13%	22.48
**Arcface**	24.72	89.19%	24.25	87.65%	16.43
**Centerface**	25.61	86.95%	25.24	83.48%	5.81
**Our**	25.59	94.18%	25.08	91.02%	3.48

It can be seen from Table 5 that all models show different performances on the self-constructed dataset and the public VeRi776 dataset, but our model maintains excellent comprehensive performance in both scenarios: on the self-constructed dataset, although CenterFace achieves the highest FPS of 25.61, its ACC is only 86.95% which fails to meet task requirements, while our model achieves the highest ACC of 94.18% with an FPS of 25.59 (only 0.02 lower than CenterFace) and the smallest Params of 3.48M, realizing a balance between accuracy, real-time performance and lightweight; on the public VeRi776 dataset where all models have lower overall ACC, our model still stands out with an ACC of 91.02% (3.37% higher than the second-ranked ArcFace and 7.54% higher than CenterFace), an FPS of 25.08 (slightly lower than CenterFace but far higher than traditional models like FaceNet) and unchanged lightweight advantage. Comprehensively considering FPS, ACC, lightweight and cross-dataset generalization, our model outperforms all baseline algorithms and is the optimal choice for practical tunnel vehicle tasks.

### 3.5 Visualize experiment

To provide demonstrative validation of our model’s vehicle re-identification capabilities, we conducted a visual experiment using images of three distinct vehicles at tunnel entry/exit points.

**Fig 12:**
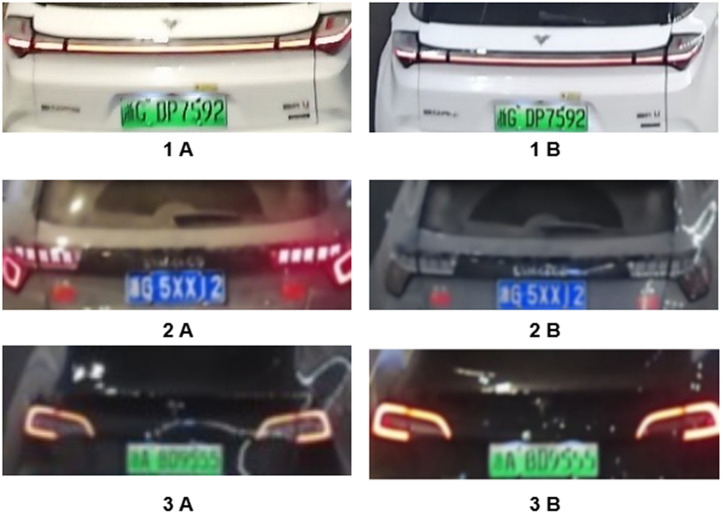
Vehicle images used in the visualization experiment.

In this experiment, the evaluation index is the Euclidean distance, and its scoring formula is shown in Equation (23) as follows [5]:


$$d=\sqrt{\sum_{i=1}^{n}{\left(x_{i}-y_{i}\right)}^{2}}$$ (23)

where d represents the distance score between two vehicles, n is the dimension of vehicle features output by the model, and $x_{i}$ and $y_{i}$ are the coordinates of feature vectors in a certain dimension, respectively. It can be seen from the formula that the Euclidean distance measures the distance between two sets of high-dimensional feature vectors in the high-dimensional space. A smaller value indicates a higher similarity between vehicles. In this paper, 1.15 is used as the threshold to measure whether the vehicles are the same.The re-identification performance is summarized in Fig 12 and Table 6 below.

**Table 6:** Visualize a table of experimental results.

Image	1 A	2 A	3 A
**1 B**	0.79	1.37	1.38
**2 B**	1.43	0.82	1.22
**3 B**	1.31	1.26	0.49

It can be seen from Table 6 above that the vehicle images of the three selected entrance-exit pairs are well distinguished under the classification of our model. With a threshold of 1.15, the Euclidean distances of the same vehicles are as small as possible (ranging from a minimum of 0.49 to a maximum of 0.79), while the Euclidean distances of different vehicles are all greater than the threshold of 1.15. This fully proves that our model has excellent vehicle re-identification capability and can meet the task requirements.

## 4. Conclusion

This study establishes a tunnel-specific vehicle detection and re-identification framework by integrating the lightweight YOLOv11n object detector with an enhanced vehicle re-identification system, with supplementary insights into dataset construction and YOLO model comparison to further validate the framework’s effectiveness. First, a dedicated tunnel vehicle dataset was built using surveillance videos from two cameras at the entrance and exit of China’s Pingyanshan Tunnel: after initial screening of 14,332 images, manual filtering removed duplicates, obstructed, and out-of-time-period samples to obtain 9,458 preliminary cleaned images (truck-to-car ratio ~1:15); targeted data augmentation (gradient attenuation for uneven lighting, Gaussian blur for camera jitter simulation) was then applied, expanding the final dataset to 12,000 images, which was partitioned into training/validation/test sets at 8:1:1 with no overlapping vehicle instances across subsets. For detection, a comparison of nano versions of YOLOv5, YOLOv8, YOLOv10, and YOLOv11 on this dataset showed YOLOv11n’s superiority: it achieved 39.5 baseline mAP (4.2% higher than YOLOv10n), 1.5ms GPU inference speed, 98.63% mAP and 4.12ms speed on the custom dataset, and 23.1% fewer parameters than YOLOv8n, with 242 fps inference speed far exceeding the 30 fps of tunnel surveillance cameras. A license plate localization module was also integrated to extract standard rear-view images, eliminating redundant information. For re-identification, the improved FaceNet (MobileNetV3 backbone + CA module + joint loss function of IoU-based hard triplet mining and Center Loss) achieved 94.18% ACC at 25.43 fps, with 0.81 GFLOPs and 3.51M Params—compared to baseline FaceNet, MobileNetV3 increased FPS by 6.31% and reduced GFLOPs/Params by 71.58%/84.73%, CA improved ACC by 0.58%, and the joint loss enhanced ACC by 1.19%. Ablation experiments confirmed the integrated model’s optimal performance; ROC analysis showed its AUC (97.26%) was 2.44% higher than original FaceNet (94.82%); comparative experiments on the self-constructed dataset and public VeRi776 dataset outperformed baseline methods (original FaceNet, CosFace, ArcFace, CenterFace) in accuracy and lightweight properties; visualization experiments (Euclidean distance threshold = 1.15) verified correct entry-exit vehicle matching, with same-vehicle distances (0.49–0.79) below the threshold and different-vehicle distances exceeding it.

The proposed framework adopts a nested architecture of YOLOv11n (2.6M parameters) and the improved re-identification model (3.51M parameters, 0.81 GFLOPs), specifically designed for embedded/edge device deployment (e.g., Jetson series, Raspberry Pi). Among these, the Jetson Nano was selected for validation due to its wide application, cost-effectiveness (low power consumption, strong compatibility) in intelligent transportation edge scenarios: the framework’s total parameter count ≤ 6.2M and low computational complexity avoid issues like insufficient computing power or memory overflow on edge devices. It also supports adaptation to lower-performance devices (e.g., Raspberry Pi) via engineering measures such as simplified post-processing, eliminating traditional models’ reliance on high-performance servers and enabling localized processing at tunnel sites with weak network signals and limited bandwidth—highlighting significant practical applicability and engineering implementation potential.

However, the proposed algorithm has limitations: it is only applicable to tunnel scenarios, and its performance is highly dependent on the placement position and angle of surveillance cameras. If the camera setup deviates from the downward-angled configuration (e.g., horizontal viewing angles, excessively elevated heights), it may lead to incomplete extraction of vehicle discriminative features (such as missing details of headlights, license plates, or body contours), thereby reducing re-identification accuracy and failing to meet the practical monitoring requirements of non-standard camera deployment scenarios.

Future work will focus on three directions to advance intelligent transportation systems toward higher autonomy: first, exploring multi-modal data fusion techniques, such as integrating LiDAR point cloud data with visual images to compensate for the limitations of single visual data in low-light, severe occlusion, or extreme weather conditions; second, investigating cross-tunnel transfer learning to enhance the model’s generalization ability across different tunnel environments (e.g., varying lengths, lighting systems, or traffic volumes) without the need for large-scale dataset reconstruction for each tunnel; third, developing advanced feature distillation techniques to further compress the model size while preserving key discriminative features, improving its deployment flexibility on more resource-constrained edge devices.

## Supporting information

S1 Data (ZIP)
