# Supplementary figures and images for: A lightweight tunnel vehicle re-ldentification model based on YOLOv11n and FaceNet
Source: PLoS One. 2025 Dec 30;20(12):e0339450. doi: 10.1371/journal.pone.0339450 (PMC12752996; doi:10.1371/journal.pone.0339450)

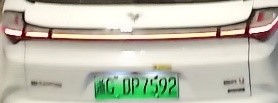

Supplement: S1 Data — (ZIP) [file pone.0339450.s001.zip › The dataset used for experimental validation/1.jpg]

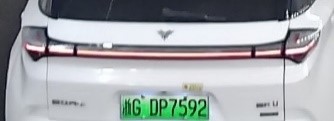

Supplement: S1 Data — (ZIP) [file pone.0339450.s001.zip › The dataset used for experimental validation/2.jpg]

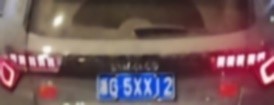

Supplement: S1 Data — (ZIP) [file pone.0339450.s001.zip › The dataset used for experimental validation/3.jpg]

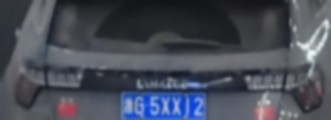

Supplement: S1 Data — (ZIP) [file pone.0339450.s001.zip › The dataset used for experimental validation/4.jpg]

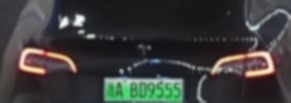

Supplement: S1 Data — (ZIP) [file pone.0339450.s001.zip › The dataset used for experimental validation/5.jpg]

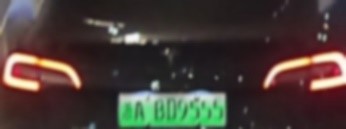

Supplement: S1 Data — (ZIP) [file pone.0339450.s001.zip › The dataset used for experimental validation/6.jpg]
